# States with fewer criminalizing immigrant policies have smaller health care inequities between citizens and noncitizens

**DOI:** 10.1186/s12889-020-09525-4

**Published:** 2020-10-15

**Authors:** Maria-Elena De Trinidad Young, Hiram Beltrán-Sánchez, Steven P. Wallace

**Affiliations:** 1 Department of Public Health, School of Social Sciences, Humanities, and Arts, University of California, Merced, USA; 2 Department of Community Health Sciences, Fielding School of Public Health, University of California, Los Angeles, USA

**Keywords:** Immigration, Citizenship, Health care access

## Abstract

**Background:**

In the last thirty years, major shifts in immigrant policy at national and state levels has heightened boundaries among citizens, permanent residents, and those with other statuses. While there is mounting evidence that citizenship influences immigrant health care inequities, there has been less focus on how policies that reinforce citizenship stratification may shape the extent of these inequities. We examine the extent to which the relationship between citizenship and health care inequities is moderated by state-level criminalization policies.

**Methods:**

Taking a comparative approach, we assess how distinct criminalization policy contexts across US states are associated with inequitable access to care by citizenship status. Utilizing a data set with state-level measures of criminalization policy and individual-level measures of having a usual source of care from the National Health Interview Survey, we use mixed-effects logistic regression models to assess the extent to which inequities in health care access between noncitizens and US born citizens vary depending on states’ criminalization policies.

**Results:**

Each additional criminalization policy was associated with a lower odds that noncitizens in the state had a usual source of care, compared to US born citizens.

**Conclusion:**

Criminalization policies shape the construction of citizenship stratification across geography, such as exacerbating inequities in health care access by citizenship.

## Background

In the US, immigrants who lack citizenship face significant barriers to accessing health care [1]. Citizenship constitutes a system of social stratification that inequitably positions individuals in a hierarchy of social and legal belonging - from US born citizens to naturalized citizens to those with various documentation statuses (e.g., lawful permanent residents, temporary resident statuses) to those who are undocumented [2]. Research shows a pattern in which inequities in access to health care align with the hierarchy of citizenship statuses. Undocumented immigrants, for example, are less likely to have insurance, a usual source of care, and receipt of timely preventative services compared to documented immigrants and US born citizens [3–7]. Those with temporary statuses, such as Deferred Action for Childhood Arrivals, similarly, are ineligible for federally-funded public insurance and face institutional barriers to establishing a source of care [8]. While more likely to have access to care than the undocumented, lawful permanent residents (i.e. green card holders) also face barriers compared to those with citizenship [9]. In contrast, naturalized citizens tend to have similar levels of access to care as US born citizens [10].

Citizenship status, while a factor in shaping access to care, is not an innate, individual-level characteristic. Rather, citizenship status reflects immigrants’ position within the nation’s ever-changing structure of rights and social belonging. A significant factor in shaping that structure are immigrant policies that determine the rights, protections, and access to resources granted to immigrants based, specifically, on their citizenship status. The 1996 federal passage of the Personal Responsibility and Work Opportunity Reconciliation Act and the Illegal Immigration Reform and Immigrant Responsibility Act launched a “devolution” of policy-making authority. These laws transferred policy-making authority, such as determining eligibility based on citizenship status or collaborating with immigration enforcement, from the federal government to state governments [11]. Currently, states have the discretion to shape the social and legal incorporation of noncitizens. Immigrant policies can contribute to noncitizens’ barriers to health care – from being shut out of eligibility for insurance to not feeling safe from discrimination in health care settings [1]. This suggests that the legal, social, and material barriers to health care that noncitizens face will differ based on a state’s composition of policies, above and beyond any individual policy.

In recent years, state policy makers have increasingly enacted restrictive immigrant policies [12]. We refer to these as criminalization policies as they constitute a punitive turn in treatment towards noncitizens that function to criminalize day-to-day activities in public and institutional spheres through surveillance and policing, and by linking immigration enforcement with the criminal justice system [11, 13, 14]. Mechanisms of criminalization likely shape the nature of citizenship stratification in each state, influencing the extent of inequities in access to health care [15]. First, criminalization policies regulate the permanence and authorization of immigrants’ presence in the country. For example, in states with policies that authorize local law enforcement to collaborate with immigration enforcement, noncitizens face a greater risk of arrest or deportation [13]. Second, criminalization policies are also state-level criminal justice laws (e.g., sentencing for minor crimes) that intersect with immigration laws. For example, a sentence of 1 year or more for specific crimes (e.g. misdemeanor theft) can render a green card holder ineligible for naturalization or subject to deportation under the federal definition of “aggravated felony.” [16, 17] Finally, criminalization policies extend surveillance and verification of status, such as through state policies requiring verification of work authorization or requiring that law enforcement check a person’s legal status [18].

Individual criminalization policies do more than restrict rights of individuals, they contribute to an overall context which shapes the legal and social position of all noncitizens. The impact of criminalization policies extends beyond individuals or groups who are directly targeted by each policy (e.g., individuals in the criminal justice system, the undocumented who lack work authorization). By selectively targeting some immigrants who may be perceived as socially “undesirable”, these policies stigmatize all noncitizens as criminal threats and reinforce their vulnerability for deportation among all noncitizens [19–21]. At the extreme, for those who are directly targeted, contact with law enforcement, the criminal justice system, or situations with required legal status verification, can result in contact with the immigration enforcement system. Even at the lesser extreme, for those who simply live their lives in contexts of criminalization policies, the mechanisms of criminalization make noncitizens’ daily lives subject to the potential of near continuous immigration surveillance.

States with more criminalization policies, such as those that we identify in Table 1, likely have contexts that exacerbate citizenship stratification, with implications for health care inequities. To understand how health care inequities are related to citizenship stratification, we examine the association between citizenship status and access to care in the context of state-level criminalization policies. Specifically, do criminalization policies moderate the relationship between citizenship status and access to health care? We use the likelihood of having a usual source of care as an indicator of health care access and of immigrants’ long-term ability to obtain timely receipt of preventative services, improve management of chronic conditions, and reduce emergency department use [22, 23]. We first test the association between citizenship status and access to care, hypothesizing that, consistent with the existing literature, noncitizens will be less likely to have a usual place where they receive health care compared to US born citizens. We then assess states’ overall context of criminalization policies, based on a count index of the policies (Table 1) that can create a context of surveillance, policing, and enforcement of noncitizens. We test the association between the interaction of criminalization policies and citizenship status and usual source of care. We hypothesize that criminalization policies will be a significant moderator, such that access to health care between noncitizens and US born citizens varies depending on states’ criminalization policy contexts.

**Table 1:** State-level criminalization policies

Area of policy	Indicator of policy
**POLICING POLICY**	Does the state authorize law enforcement to fully collaborate with federal immigration authorities?^a^
	Does the state require or allow law enforcement to verify individuals’ legal status at the time of a stop or arrest?^b^
**CRIMINAL JUSTICE POLICY**	Does the state sentence certain criminal offenses at at least 365 days (e.g., federal immigration criteria for “aggravated felony”)?^b^
**VERIFICATION AND ID POLICY**	Does the state require a social security number to obtain a driver’s license?^c^
	Does the state comply with REAL ID?^d^
	Does the state mandate employers use E-Verify?^e^

**Data sources accessed to determine if each state had enacted policy by December 31, 2013**

^a^Immigrant Legal Resource Center - Immigration Detainer Map. Avialable at: http://www1.ilrc.org/detainer/detainermap.html

^b^César Cuauhtémoc García Hernández. *Crimmigration Law.* American Bar Association. 2015 and author’s review of state statutes

^c^National Council of State Legislatures, Immigrant Policy Project Available at http://www.ncsl.org/research/immigration/states-offering-driver-s-licenses-to-immigrants.aspx

^d^National Council of State Legislatures, available at http://www.ncsl.org/documents/standcomm/sctran/REALIDComplianceReport.pdf

^e^National Council of State Legislatures, State E-Verify Action: available at http://www.ncsl.org/research/immigration/everify-faq.aspx

## Methods

We created a data set with person-level measures of citizenship and health care access, and state-level measures of criminalizing immigrant policy. A multi-level multivariable analysis assesses whether or not the association between citizenship status and having a usual source of care was moderated by states’ level of criminalization policy.

### Data sources

#### Person-level data

Data on health outcomes and socio-demographic characteristics of individuals came from pooled 2014 and 2015 National Health Interview Survey (NHIS) public and restricted Person and Household files. The NHIS collects data on **non-institutionalized households in the United States** [24]**.** The restricted files contain data on state of residence and immigration-related characteristics. We included individuals who were ages 18–64 **and identified as Latino, White, Black, or Asian for a total of** 52,562 respondents.

#### State-level data

For data on state immigrant policy, we used an existing policy dataset that categorized states and the District of Columbia based on the presence of six criminalization policies (Table 1). For each policy, a state was coded as 1 - Yes, having the policy or 0 - No, not having the policy. Information on each state’s policy was identified through a systematic review of secondary data sources on state legislation, administrative regulations, or court rulings (BLINDED). States were coded as having a policy if it was enacted any time before December 31, 2013, a date aligning with the end point of a period of extensive new state-level policy activity [25]. The data set also included information on whether or not states had inclusive policies that granted eligibility or rights in health and social service, higher education, labor and employment, and language sectors (Additional file 1).

### Measures

#### Health care access

For access to care, we categorized individuals based on self-report of having a usual place where they receive care when they are sick (Yes = 1, No = 0).

#### Citizenship status

Using NHIS immigration questions, we classified individuals as being a noncitizen, a naturalized citizen, or a US-born citizen. We used noncitizens as the reference group to assess how the group at the lowest rank of the citizenship hierarchy compares with the two groups of US citizens.

#### Level of criminalization

Using the policy dataset, we tallied the number of criminalization policies present in each state (possible range = 0–6, observed range = 1–6). This measure was used as a continuous variable, with increasing values representing a greater level of criminalization. (Additional file 2 presents state scores).

#### Individual-level covariates

Race/ethnicity was coded using the NHIS self-reported questions about Hispanic origin and race. Respondents were asked if they are of Hispanic or Latino origin (e.g., Mexican, Central American, etc.). They were then asked to report the race(s) with which they identify (e.g., White, Black, Asian). From the two questions, we categorized individuals as Latino (of any race), Non-Latino White, Non-Latino Black, and Non-Latino Asian/Pacific Islander (API). Additional covariates included age (continuous years), sex (1 = female, male = 0), education (1 = high school graduate; no high school diploma = 0), employment (1 = employed; unemployed or not in the labor force = 0), marital status (1 = married; separated/divorced, widowed, never married = 0), insured for all of last year (1 = insured full year; uninsured some or all of past year = 0), speaks English only or well (1 = yes, 0 = no), and self-reported health (1 = Excellent, 2 = Very good, 3 = Good, 4 = Fair, 5 = Poor).

#### State-level covariates

To assess if the variation in the outcomes was influenced independently by criminalization policies—and not other state characteristics—we included state-level covariates that captured demographic and policy conditions. We included the index of inclusion policy, a tally of each state’s total number of inclusion policies (observed range = 2–11), to account for the potentially countervailing influence that these policies may have on immigrants’ access to care (Additional file 2). To account for contextual demographic factors, we controlled for the percent of the state that was foreign-born [26]. To account for political polarization regarding immigration issues, we controlled for the percent of the electorate who voted Republican in the 2012 presidential election [27], the closest presidential election to the date of NHIS survey collection in 2014–15.

### Statistical analysis

We conducted analyses with Stata 14 software through the US Census Bureau Restricted Data Center at [BLINDED]. We conducted descriptive analysis to assess the means and distributions of each variable across citizenship status. To assess variations in usual source of care across immigrant policy contexts, we used unweighted mixed-effects logistic regression models with a random effect for state of residence. We used mixed-effects modeling due to the inclusion of state-level variables and used unweighted models as the NHIS is not representative at the state-level and does not have weights for this level of analysis. The first model tested the main effect associations between having a usual source of care and citizenship, controlling for criminalization policy and individual- and state-level covariates. Insurance status and self-reported health were significant, but neither had a notable impact on the magnitude of the coefficient for citizenship status. For parsimony, we retained only insurance status. We also retained only state-level covariates that contributed significantly to the outcome, which resulted in omitting the state-level covariate for percent of the state that was foreign-born. To assess if the association between having a usual source of care and citizenship varied by the level of criminalization policy, we estimated a mixed-effects logistic regression model testing the association between having a usual source of care and a two-way interaction of the level of criminalization policy and citizenship status, including all lower-order effects and covariates. We used a chi-squared test to assess the significance of the interaction.

## Results

### Sample characteristics by citizenship status

About 10 % of respondents were noncitizens, 9.2% were naturalized citizens, and 80% were US-born citizens (Table 2). Overall, 83% percent of the sample reported having a usual source of care. Respondents lived in states with a mean of 3.5 criminalization policies, indicating that, on average, noncitizens across states live in contexts made up of numerous policies. This mean did not vary significantly by citizenship status.

**Table 2:** Unweighted means and distributions by citizenship status for sample socio-demographic characteristics

	Noncitizen	Naturalized Citizen	US Born Citizen	All
	***n*** = 5687	***n*** = 4827	***n*** = 42,048	***n*** = 52,562
	%	%	%	%
**Citizenship Status**				
Noncitizen				10.8
Naturalized Citizen				9.2
US Born Citizen				80.0
**# of criminalization policies (range: 1–6)**	3.2 ± 0.01	3.3 ± 0.01	3.6 ± 0.005	3.5 ± 1.1
**Has usual source of care**				
Yes	67.3	84.7	84.9	83.0
No	32.7	15.3	15.1	17.0
**Race**				
Latino	65.1	43.2	10.1	19.1
White	8.3	18.8	72.0	60.1
Black	6.3	11.3	15.9	14.5
Asian	20.3	26.8	2.0	6.3
**Sex**				
Male	48.1	44.1	45.9	46.0
Female	51.9	55.9	54.1	54.1
**Age**	38.3 ± 0.1	44.4 ± 0.2	42.0 ± 0.07	41.8 ± 0.13
**High school graduate or higher**				
Yes	59.4	83.9	91.3	87.1
No	40.6	16.1	8.7	12.9
**Currently working**				
Yes	72.5	80.5	78.7	78.2
No	27.5	19.5	21.3	21.8
**Currently married**				
Yes	56.1	58.1	44.4	47.0
No	43.9	41.9	55.6	53.0
**Has health insurance**				
Yes	87.8	92.5	92.5	92.1
No	12.2	7.5	7.5	7.9
**Speaks English Well**				
Yes	54.7	84.9	99.7	93.4
No	45.3	15.1	0.3	6.6
**# of inclusion policies (range: 2–11)**	7.0 ± 0.04	6.8 ± 0.04	5.2 ± 0.01	5.7 ± 2.9
**% state voted Republican 2012**	0.45 ± 0.00	0.44 ± 0.00	0.49 ± 0.00	0.48 ± 0.1

Notes

Source: National Health Interivew Survey, 2014–2015, Latino, White, Black, and Asian adults ages 18–64

*sd* Standard deviation

Across citizenship statuses, there were notable differences in having a usual source of care and in state- and individual-level characteristics. Only 67.3% of noncitizens reported having a usual source of care, compared to about 85% of both naturalized and US born citizens. About two-thirds of noncitizens were Latino (65.1%) and one-fifth (20.3%) were Asian or Pacific Islander (API). Among naturalized citizens, these fractions differed, with 43.2% Latino and 26.8% API. In contrast, almost 75% of the US-born citizen population was non-Latino White. Compared to their citizen counterparts, noncitizens were younger, had lower rates of high school graduation, had lower rates of having health insurance, and lower levels of English language fluency. Noncitizens and naturalized citizens lived in states with higher mean number of inclusion policies (7.0) than US-born citizens (5.2). Noncitizens also lived in states with lower mean proportions of Republican voters.

### Associations between usual source of care, citizenship status, and criminalization policy

Mixed effects models predicting a usual source of care are shown in Table 3. Model A includes all covariates while Model B adds an interaction between citizenship status and the number of criminalization policies. Results from Model A show that the odds of having a usual source of care are higher among citizens than noncitizens. For example, US born citizens had 1.45 the odds and naturalized citizens had 1.28 the odds of having a usual source of care compared to noncitizens, net of the effects of criminalization policy and individual and state-level covariates. The number of criminalization policies was associated with 0.90 the odds of having a usual source of care; indicating that each additional criminalization policy was associated with 10% lower odds of having a usual source of care, independent of citizenship status and other individual-level characteristics. In addition, compared to Latinos, non-Latino Whites and Asians had lower odds of having a usual source of care, net of other covariates, but not Blacks. Women and those who had a high school degree, were currently married, had health insurance, and spoke English well had significantly higher odds of having a usual source of care. Those who were currently working had lower odds of having a usual source of care. At the state-level, a higher number of integration policies was associated with a slight reduction in the odds of having a usual source of care. Each additional integration policy (e.g., Medicaid for undocumented children or in-state university tuition for undocumented youth) was associated with a 3% decrease in the odds of having a usual source of care, regardless of citizenship.

**Table 3:** Unweighted mixed-effects logistic regression model of the association between having a usual source of care and (A) citizenship status and (B) the interaction of citizenship status and criminalization policy

	Model A		Model B	
	OR	***p***-value	OR	***p***-value
**Citizenship**				
US Born	1.45	< 0.05	1.20	0.1
Naturalized	1.28	< 0.05	1.60	< 0.05
Noncitizen	ref		ref	
**# Criminalization Policies**	0.91	< 0.05	0.90	< 0.05
**Race/Ethnicity**				
White	0.91	0.04	0.90	0.02
Black	0.92	0.1	0.91	0.8
Asian	0.82	< 0.05	0.81	< 0.05
Latino	ref		ref	
**Age**	1.03	< 0.05	1.03	< 0.05
**Gender**				
Female	2.10	< 0.05	2.10	< 0.05
Male	ref		ref	
**High school graduate or higher**				
Yes	1.11	< 0.05	1.12	< 0.05
No	ref		ref	
**Currently working**				
Yes	0.90	< 0.05	0.90	< 0.05
No	ref		ref	
**Currently married**				
Yes	1.46	< 0.05	1.48	< 0.05
No	ref		ref	
**Has health insurance**				
Yes	6.39	< 0.05	6.40	< 0.05
No	ref		ref	
**Speaks English Well**				
Yes	1.13	0.04	1.10	0.04
No	ref		ref	
**# Inclusion Policies**	0.97	0.04	0.97	0.03
**Percent of state that voted Republican in 2012**	0.40	< 0.05	0.40	< 0.05
Citizenship X Criminalization policy*				
NaturalizedXCriminalization Policy			0.93	0.1
US BornXCriminalization Policy			1.06	0.05
NoncitizenXCriminalization Policy			ref	
**Intercept**	0.32	< 0.05	0.36	< 0.05
**State random intercept**	1.11		1.10	

Notes

Source: National Health Interivew Survey, 2014–2015, Latino, White, Black, and Asian adults ages 18–64 (*n* = 51,581)

*Interaction term statistically significant at *p* < 0.05

Model B presents the mixed effects logistic regression model testing the association between usual source of care and the interaction of citizenship status and the number of criminalization policies, net the effects of the individual- and state-level covariates. A chi-squared test of the overall interaction (i.e., the multi-degree of freedom interaction) indicated that the level of criminalization policy moderated the relationship between citizenship status and usual source of care. The odds of having a usual source of care varied significantly (*p* < 0.05) by citizenship status across the number of criminalization policies. The interaction indicates US born citizens had higher odds of having a usual source of care than noncitizens for every increase in criminalization policies. Additional file 3 shows the predicted probabilities of having a usual source of care for each of the three citizenship status categories in states with 0 criminalization policies and states with 6 criminalization policies. The pattern indicates that the difference between noncitizens and US born citizens in the predicted probabilities of having a usual source of care was greatest in the states with the highest level of criminalization policy. Further, noncitizens in these states had the lowest predicted probability of a usual source of care compared to noncitizens in states with lower levels of criminalization policy. There was no significant variation in having usual source of care between noncitizens and Naturalized citizens by criminalization policies. The overall model indicates that an increase in the level of criminalization policies was associated with a significant population-level decline in health care access and that the difference between noncitizens and citizens increased with greater numbers of criminalization policy.

## Discussion

This study sought to examine inequities in access to care by citizenship in the context of criminalization policies. Consistent with existing evidence that links citizenship stratification with access to health care [28], noncitizens in this study were less likely to report having a usual source of care than either naturalized or US born citizens. In the last 30 years, the federal government, states, and localities have increasingly enacted criminalization policies in the areas of policing, criminal justice, and identification and legal status verification [11]. In this study, individuals lived in states with an average of over three criminalization policies, indicating that across the US many noncitizens seek health care in contexts of surveillance, policing, and enforcement. Examining the inequity in health care between noncitizens and US born citizens, we found that states with more criminalization policies had greater inequities in access. Each additional criminalization policy in individuals’ state of residence was associated with a lower likelihood that noncitizens had a usual source of care, compared to US born citizens. These findings indicate that citizenship inequities in health care access are not uniform; rather, states’ context of criminalization policy are related to the extent of health care inequity between citizens and noncitizens. Below we discuss the possible relationship between criminalization policy and health care and how this informs understanding of citizenship stratification and health care equity, as well as future research directions.

Our findings suggest that noncitizens in a more criminalizing state may face greater barriers to health care access that noncitizens in a less criminalizing state. A growing body of literature has linked restrictive, anti-immigrant policies to barriers in access to care [29]. Qualitative studies of local enforcement policies found that immigrants of different noncitizen statuses, fearing deportation due to federal and local immigration enforcement policies, avoided seeking health care [30–32]. Residents felt that enforcement could have a negative impact on child health, their mobility (e.g. driving without a driver’s license), and their ability to access legal services; in particular, there was a perception that enforcement policies condoned racial profiling of Latinos. These studies were conducted in diverse immigrant communities, where individuals possessed different legal statuses, showing that the impact of enforcement policies extends beyond undocumented immigrants and affects all immigrants’ perceptions of safety and acceptance in their communities. The current study extends the existing evidence to show that it may not be any one *single* policy that creates barriers to care but the *context* of numerous policies that produce mechanisms of policing, enforcement, and surveillance. Overall contexts of criminalization policy likely produce a range of barriers related to the legal, economic, and social environment. It is worth noting that the policies included in the measure of criminalization did not include any formal health policies, furthering strengthening the evidence that non-health policies can have an impact on immigrant health care access.

These findings also suggest that criminalization policy is related to the creation of inequality between citizens and noncitizens across US states. Criminalization policy represents the intentional decisions of policy makers and efforts of policy advocates to be punitive towards noncitizens. These policies utilize state institutions (e.g., law enforcement, courts) to reinforce noncitizens subordinate status in a range of sectors and settings, from health care to the workplace to jails [33, 34]. Further, criminalization of immigrants occurs in the context of criminalization of people of color in US. Criminalizing immigrant policy co-occurs with the trend of using punitive policy mechanisms to criminalize people of color in the US, generally [35]. In states and communities with criminalization policies, immigrant residents often report greater experiences of discrimination and policing practices that reinforce racial profiling [36, 37]. Considered from the perspective of citizenship stratification, state immigrant criminalization policies can be viewed as a marker of social inequality across the US population. In other words, states with more criminalization policies may be, on the whole, more inequitable across multiple sectors.

Our findings suggest that criminalization policies are related to the construction of citizenship stratification across geography and the related inequities in health care access by citizenship. These findings expand the research on citizenship stratification, providing empirical evidence of citizenship as a dynamic and changing social structure that inequitably positions immigrants within society. Criminalization policies constitute a broad context that, beyond the immediate impact on areas such as policing or enforcement, influences the nature of inequality among immigrants and native-born individuals. Changing, and increasingly criminalizing, policy contexts nationally and within states and localities are likely reinforcing the nation’s citizenship hierarchies.

### Limitations and future directions

The US states offer an ideal comparative case for assessing the variation in citizenship stratification and health access related to criminalization policy. In the US, individuals reside under the same set of federal laws and policies, while being exposed to different state policy contexts. The US, however, has a unique history of migration and immigrant criminalization and integration. To understand the relationships between citizenship, criminalization policy, and health, future research should examine variation in citizenship stratification at different scales: within other nations and their states, across counties or municipalities that have distinct policies, or across federated regions, such as the European Union, which share some *immigration* policies, but determine *immigrant* policies at the national level.

Second, while this study provides a descriptive assessment of these complex dynamics between citizenship, policy, and health care access it is cross-sectional, limiting any causal interpretation or understanding of the stage at which policy may have the most significant impact on health care access (e.g., policy enactment, implementation, etc.). We controlled for political factors and did not find demographic factors to be significant in our model. The study design, however, is not intended to establish a causal link between citizenship, policy, and health care access, but to present current patterns of inequities across states that have enacted varying numbers of criminalization policies. Given existing evidence, it is likely that criminalization policies have an impact on barriers to care. It is also possible, however, that states in which there are existing barriers to care for noncitizens are more likely to enact criminalizing policies. These are related dynamics and therefore, do not diminish the significance of the study findings. Future research should disentangle these directions by assessing the relationships between the factors that influence policy-making and the health care access outcomes associated with policy enactment. For example, recent studies suggest that enactment of restrictive policies in states was associated with both state’s economic insecurity (e.g., unemployment rate) and demographic change (e.g., Hispanic population change) [38]. Other studies found that that factors such as political polarization are also important [39]. Research could examine the extent to which criminalization policy is a response to economic or demographic anxieties and how these state-level dynamics influence how policies unfold for the policing, surveillance, and enforcement that noncitizens may subsequently experience.

The presence of criminalizing policies and variations in health care inequities by citizenship status reflects an overall treatment of immigrants that determines their position in legal, institutional, and social realms in a state. Measurement of state immigrant policy, however, continues to be a challenge and our measure presents some limitations that can be addressed in future studies [40]. Our measure of criminalization policy does not include an indication of the intensity of implementation, nor individuals’ experiences criminalization policy. While these approaches have been used in past studies, future studies could combine measures of the presence of policy, indicators of implementations (e.g., administrative records), and survey data on individuals’ experiences of policy. Future studies should similarly be based in a strong theoretical foundation.

Finally, citizenship status in the NHIS does not measure the legal and documentation statuses of noncitizen immigrants. If broken down by legal status groups, the most vulnerable among the noncitizen group, such as those who are undocumented or have Temporary Protected Status, may show an even greater inequality in access to health care compared to US born citizens. Future research should be conducted with data sets that include citizenship and legal statues.

## Conclusions

This study has significant implications for understanding of the factors that may shape immigrants’ access to care and to understanding of the nature of citizenship health inequities across the US. Access to healthcare constitutes a critical resource to promote and maintain well-being and is an important aspect of social inclusion. The extent to which health care is available or provided to immigrants can be considered a marker of social inclusion [28, 41]. In the US, noncitizens’ worse access to care reflects their broader social marginalization. This study suggests that criminalization policies are a distinct mechanism for reinforcing that marginalization. This study also points to the role of criminalizing immigrant policies in producing health inequity *between* states; noncitizens in the US currently live across distinct environments and face varying levels of policing, surveillance, and enforcement, shaping their access health care and the citizenship position in states. Noncitizens are facing increasing marginalization nationally, as well as in states and localities, with long-term implications for relatively healthy populations. The nature and structure of citizenship stratification may be, generally, more equitable in states where noncitizens are not as directly targeted by punitive and restrictive policies. Advancing policies that de-criminalize or buffer immigrants from federal enforcement is critical to promote immigrants’ access to care and well-being.

## Supplementary information

**Additional file 1.** State-level immigrant inclusion policies

**Additional file 2.** Number of state immigrant criminalization and inclusion policies, Enacted by December 31, 2013

**Additional file 3.** Predicted probability of having a usual source of care across levels of criminalization

## Data Availability

The dataset analyzed in the current study are not publicly available due to use of restricted data that requires approval from the National Center for Health Statistics and the Census Bureau.

## Abbreviation

NHIS: National health interview survey
