# Supplementary material for: States with fewer criminalizing immigrant policies have smaller health care inequities between citizens and noncitizens
Source: BMC Public Health. 2020 Oct 15;20:1460. doi: 10.1186/s12889-020-09525-4 (PMC7558673; doi:10.1186/s12889-020-09525-4)

Appendix C. Predicted probability of having a usual source of care across levels of criminalization

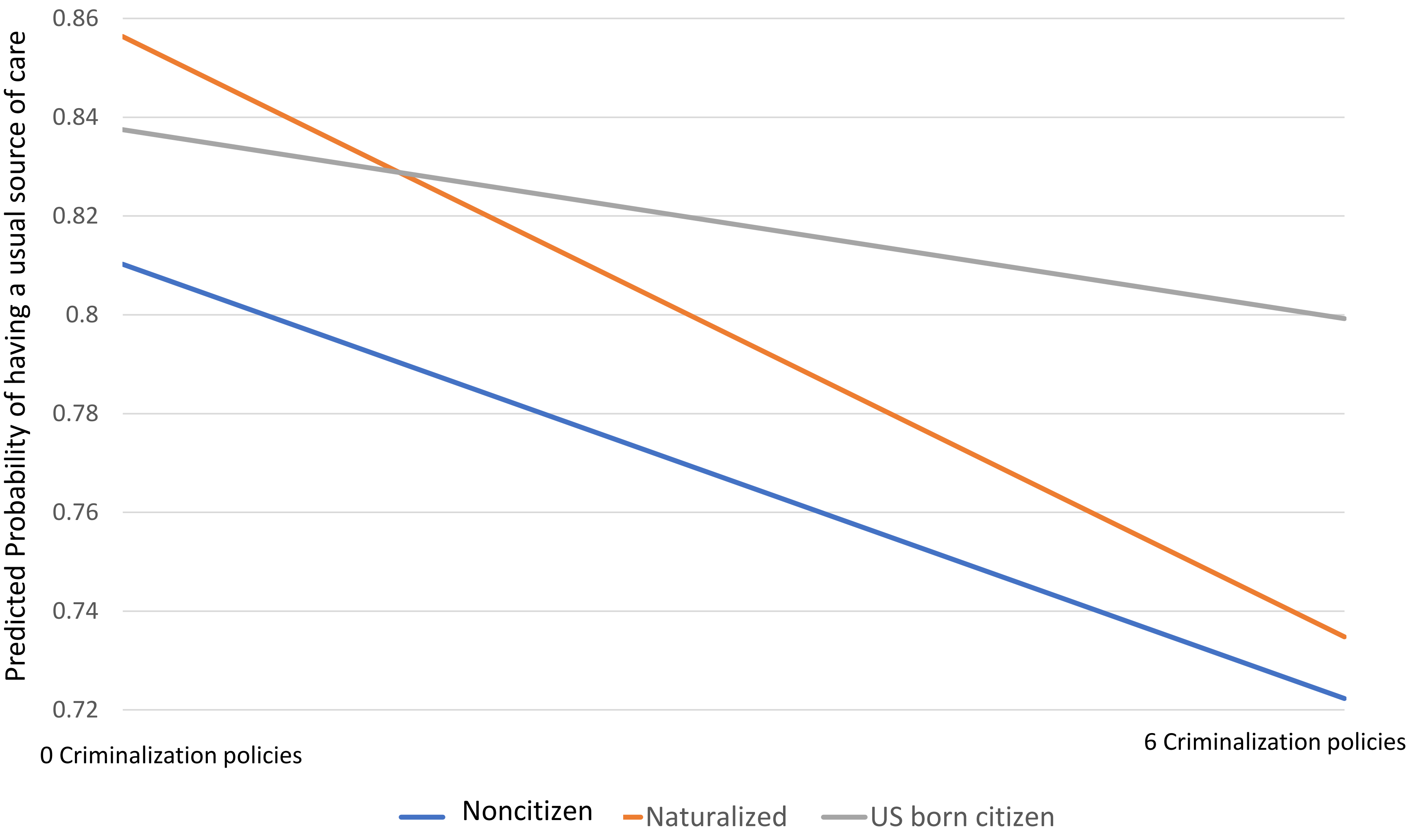

Supplement: Supplementary file 3 — Additional file 3. Predicted probability of having a usual source of care across levels of criminalization [file 12889_2020_9525_MOESM3_ESM.pdf]
